# Impact of Psychopathy on Moral Judgments about Causing Fear and Physical Harm

**DOI:** 10.1371/journal.pone.0125708

**Published:** 2015-05-20

**Authors:** Elise M. Cardinale, Abigail A. Marsh

**Affiliations:** Department of Psychology, Georgetown University, Washington, DC, United States of America; Utrecht University, NETHERLANDS

## Abstract

Psychopathy is a personality variable associated with persistent immoral behaviors. Despite this, attempts to link moral reasoning deficits to psychopathic traits have yielded mixed results with many findings supporting intact moral reasoning in individuals with psychopathic traits. Abundant evidence shows that psychopathy impairs responses to others’ emotional distress. However, most studies of morality and psychopathy focus on judgments about causing others physical harm. Results of such studies may be inconsistent because physical harm is an imperfect proxy for emotional distress. No previous paradigm has explicitly separated judgments about physical harm and emotional distress and assessed how psychopathy affects each type of judgment. In three studies we found that psychopathy impairs judgments about causing others emotional distress (specifically fear) but minimally affects judgments about causing physical harm and that judgments about causing fear predict instrumental aggression in psychopathy. These findings are consistent with reports linking psychopathy to insensitivity to others’ fear, and suggest that sensitivity to others’ fear may play a fundamental role in the types of moral decision-making impaired by psychopathy.

## Introduction

Perhaps no psychological disorder is as closely linked to immoral behavior as psychopathy. Among the core affective features that characterize psychopathy are impairments in moral emotions like empathy, remorse, and guilt [1]. These features, which are continuously distributed throughout the population rather than being limited to a distinct taxon of “psychopaths” [2–3], increase individuals’ risk for engaging in all manner of antisocial, immoral, criminal, and violent behaviors [4–8]. The question is: Why? By what mechanisms do psychopathic personality traits increase the risk for engaging in antisocial and immoral behaviors? In an effort to address this question, various moral judgment paradigms have been used to understand how psychopathy shapes perceptions of the permissibility of immoral actions. But results have been surprisingly divergent, showing that psychopathy alternately leads to minimal, mixed, or gross deficits in moral judgments [9]. We present evidence that aims to clarify this inconsistency. In three studies, we find that the effect of psychopathy on moral judgments may depend on the extent to which the judgments require the representation of the victim’s emotional distress, particularly fear, as impermissible.

Impairments in fear-based responding have long been linked to psychopathy. Fear is the emotional state associated with the anticipation of harm [10] and a variety of tasks have shown that psychopathy diminishes anticipatory fear responses. Physiologically, psychopathy has been demonstrated to reduce electrodermal activity and potentiated startle reflex during pain anticipation [11–17] despite indications that psychopathic individuals experience physical pain itself as aversive [15,18]. This suggests that anticipating an aversive outcome does not sufficiently motivate avoidance behavior in psychopathy, a hypothesis supported by findings of impaired Pavlovian conditioning and passive avoidance learning in psychopathy [14,19–21] and reduced subjective experiences of fear during threatening situations [19,22]. Psychopathy also impairs understanding others’ fear, including the recognition of fear communicated via the face, body postures, and voice [23]. This impairment appears to be most closely linked to the interpersonal and affective features of psychopathy [24] and persists even for preattentively processed stimuli [1].

These deficits are thought to result from neural dysfunction in structures associated with fear-based learning and decision-making, such as the amygdala and orbitofrontal cortex [25]. Individuals with psychopathic traits fail to exhibit typical patterns of activation in these regions during aversive conditioning and when viewing fearful facial expressions [26–29]. The ability to identify others’ fear and distress is thought to be essential to experiencing empathic concern [30]. Therefore, in individuals with high levels of psychopathic traits, deficits in fear processing may impair moral judgments about transgressions that cause victims fear [31,32]. Anecdotal evidence supports this conclusion. For example, when asked during a prison interview why he failed to empathize with his victims, one psychopathic sex offender responded, “They are frightened, right? But, you see, I don't really understand it. I've been frightened myself, and it wasn't unpleasant” [33].

That moral judgments in psychopathy are most affected when the judgments rely on representations of affective distress has played a prominent role in theories about psychopathy and morality [34,35]. Several studies have found that psychopathy affects the ability to distinguish moral violations, which primarily result in harm to a victim, from conventional violations, which primarily violate social rules or norms [35–37]. These results are interpreted as supporting the fact that psychopathy impairs the incorporation of victims’ distress into moral judgments [34,35]. However, moral/conventional reasoning tasks do not explicitly assess responses to victim distress. The moral and conventional violations described are not always clearly distinguishable in terms of the extent to which they result in victim distress, and they differ from one another in ways unrelated to victim distress [38]. Perhaps as a result, some studies fail to find clear relationships between psychopathy and the ability to distinguish moral and conventional violations [39]. One interpretation of this inconsistency is that psychopathy specifically impairs responsiveness to others’ emotional distress, leaving tasks that focus on judgments about physical harm, which is an imperfect proxy for emotional distress, relatively insensitive to this impairment. Consistent with this, other studies using trolley-type moral dilemmas, in which respondents judge the permissibility of harming innocent victims to save other lives, have failed to find consistent correspondence between moral judgments and psychopathy in institutionalized and community samples [40–42]. Again, these inconsistencies may result from the fact that these moral reasoning paradigms are not designed to assess how victim distress specifically drives moral judgments.

It is of course inherently difficult to target responses to victim distress in moral judgment paradigms. In many such tasks, judgments about the victim’s emotional response to actual or potential physical harm cannot easily be dissociated from judgments about the physical harm itself. For example, in a typical “trolley” dilemma, respondents might be asked whether it is permissible to push an innocent victim into the path of an oncoming trolley to save five bystanders. Judgments that this action is impermissible may reflect the distress that the victim would experience. Alternately, these judgments may simply reflect semantic knowledge about proscriptions against deliberately causing physical harm and the cognitive understanding that five lives are greater than one. And in fact, the results of several neuroimaging studies have now demonstrated that individuals with high versus low levels of psychopathy may engage in distinct processes to arrive at decisions in social and moral judgment paradigms [32,43–45]. Among individuals with low levels of psychopathy moral judgment paradigms engage more affectively based processes centered around the amygdala, but individuals with high levels of psychopathy appear to rely more on abstract reasoning and semantic knowledge based processes centered in frontal areas such as dorsolateral prefrontal cortex [32,44,46]. These findings typically result from separate assessments of patterns of covariance within the high-scoring and low-scoring group and reinforce the fact that, although psychopathic traits are continuously distributed in the population, the behavioral or cognitive expression of these traits may not vary continuously. Rather, discontinuities in cognitive processes may be observed in the high and low portions of the spectrum such that individuals with higher levels of psychopathic traits rely more heavily on deliberate, cognitive reasoning and less on automatic, affective processes.

In order to determine how moral judgments in psychopathy are influenced by the victim’s emotional distress, the distress must be decoupled from the physical harm itself. This has not previously been attempted: no prior study specifically compares responses to physical harm versus emotional distress in psychopathy. We endeavored to accomplish this in three studies. In Study 1, we compared judgments about causing victims physical harm versus emotional distress using two previously validated tasks, the Moral Foundations Questionnaire (MFQ), which measures judgments about causing victims harm (but not emotional distress), and the Emotionally Evocative Statements Task (EEST), which measures judgments about causing victims various forms of emotional distress (but not physical harm) [31,32]. We predicted that psychopathy scores would correspond more closely to responses about causing emotional distress (particularly fear) in the EEST than to responses about causing victims harm in the MFQ. For Study 2, we developed a novel moral judgment task aimed at testing our specific hypotheses. We generated a set of 32 moral dilemmas that dissociate judgments about causing physical harm from those about emotional distress (fear) while keeping all other features of the scenarios (such as intentionality) constant. Using this task, we assessed the correspondence between psychopathy and moral judgments about causing emotional distress versus physical harm, and in Study 3 we also assessed how responses in this task correspond to aggressive behavior measured using the Reactive and Proactive Aggression Questionnaire (RPQ).

## Study 1

### Participants

Forty males (*N* = 15) and females (*N* = 25), ages 18–38 years (*M* = 20.57, *SD* = 3.16) were recruited from Georgetown University and the surrounding community and received monetary compensation for their participation. No participants were excluded from analysis. Written consent was obtained from all participants prior to participation in the study. The Georgetown University Institutional Review Board approved all procedures and all participants provided written informed consent before participating.

### Method

The Emotional Evocative Statements Task (EEST) was presented on a desktop computer using the program Superlab. The 100 statements in the EEST were created to selectively evoke one of five emotions: anger, disgust, fear, happiness, or sadness [31,32]. Sample statements include: anger (“You are a disgrace”), disgust (“I never wash my hands”), fear (“You better watch your back”), happiness (“I bought you a present”), and sadness (“I don’t want to be friends anymore”). Participants viewed these statements in randomized order three times. In the first viewing, participants rated the extent to which it would be morally acceptable to make each statement to another person. Response options were: 1 = Never Acceptable, 2 = Rarely Acceptable, 3 = Usually Acceptable, 4 = Always Acceptable. In the second and third viewings respectively, participants identified which emotion another person would likely feel if someone were to make the statement to them, and which emotion they themselves would feel were someone to make the statement to them in a forced-choice paradigm. Participants next completed the Moral Foundations Questionnaire (MFQ), which assesses the relevance of abstract concepts that include Harm, Fairness, Ingroup, Authority, and Purity [39]. Participants also completed a 5-point political orientation scale (1 = Very Liberal, 3 = Middle of the Road, 5 = Very Conservative) to confirm the validity of responses to the MFQ. It has been previously shown that political conservatives accord higher importance to Authority and Purity domains whereas political liberals are more concerned with the domains of Harm and Fairness [47]. We next measured psychopathy using the Psychopathic Personality Inventory-Revised (PPI-R) [48]. The PPI-R is a 154-item self-report measure that measures psychopathic traits dimensionally, in accordance with the consensus that psychopathy is continuous rather than taxonomic in structure [2]. The PPI-R shows similar relations with criterion measures as file-review based measures of psychopathy, notably the PCL-R, which is used to assess psychopathy in institutionalized samples [49]. The PPI-R and its predecessor have been successfully used in previous investigations of social and moral decision-making in psychopathy [31,32,50–52]. Finally, participants completed a demographic questionnaire. All measures were self-timed.

### Results

Participants’ moral judgments in response to each of the 5 emotional categories on the EEST were calculated separately by computing the mean of their judgments for the 20 items in each category. Mean judgments were comparable to those observed in previous studies using this instrument: anger (*M* = 1.60 *SD* = 0.35), disgust (*M* = 2.03, *SD* = 0.43), fear (*M* = 1.62, *SD* = 0.32), happiness (*M* = 3.83, *SD* = 0.25), and sadness (*M* = 2.23, *SD* = 0.45). We next calculated subscale scores on the MFQ, which ranged from: 6–25 (*M* = 17.18, *SD* = 4.66) for Authority, 6–29 (*M* = 22.28, *SD* = 3.99) for Fairness, 11–28 (*M* = 22, *SD* = 3.69) for Harm, and 2–22 (*M* = 14.60, *SD* = 5.62) for Purity. Political Orientation scores ranged from 1–5 (*M* = 2.69, *SD* = 0.92). Finally, we compiled participants’ PPI-R scores, which were approximately normally distributed (kurtosis = -0.34, *SE* = 0.733; skewness = -0.27, *SE* = 0.374) with a mean of 274.59, (*SD* = 35.66), a median of 274, and a range of 184–337. Reliability of the PPI-R was acceptable (α = .68). These values are comparable to those observed previously [32] and indicate that our sample contained sufficient variability to support the planned analyses.

Following our prior approach [31] we first conducted a multiple regression analysis in which total PPI-R scores were the dependent variable and EEST scores for moral permissibility responses for the five categories of moral judgments were the predictor variables. This enabled us to assess how responses in the EEST predicted psychopathy (because this study is correlational, the term “prediction” refers here to statistical prediction rather than temporal causation). Results of an analysis of multicollinearity among the five emotion factors of the EEST found acceptable variance inflation factor values (*M* = 2.53) and tolerance values (*M* = 0.49) (Table 1). We found the overall model to significantly predict psychopathy, *R* ^*2*^ = .28, *F*(5, 34) = 2.59, *p* = .043 (Table 1). Directly replicating previous findings, we confirmed that psychopathy is best predicted by judgments about the permissibility of causing others fear, *β* = .59, *t*(34) = 2.34, *p* = .026. Raw correlations confirmed this relationship between psychopathy and moral judgments about causing fear, *r*(38) = .35, *p* = .026, as did the results of a median split for which responses to fear-evoking items were compared for participants with high and low psychopathy scores, *t*(38) = 2.00, *p* = .053, *r* = -.30, at the trend level. Regression results also indicated that psychopathy was predicted by judgments about causing sadness, *β* = -.51, *t*(34) = 2.03, *p* = .050, however the bivariate correlation did not support a strong relationship between these variables, *r*(39) = .05, *p* = .747. No other emotion category was a significant predictor of psychopathy scores.

**Table 1:** Regression model with moral evaluations of causing each emotion factors from the Emotionally Evocative Statements Task (EEST) predicting psychopathy scores.

	Coefficients		Multicolinearity	
Predictor Variables	*β*	*t*(39)	VIF	Tolerance
**Anger**	.39	1.33	3.90	.26
**Disgust**	-.25	-1.34	1.68	.60
**Fear**	.59	2.34*	2.97	.34
**Happiness**	-.10	-0.68	1.10	.91
**Sadness**	-.51	-2.03	3.00	.33

Overall model: *R* ^*2*^ = .28, *F*(5, 34) = 2.59, *p* = .043.

* *p* < .05

We next conducted a parallel analysis across moral domains of the MFQ. Total PPI-R scores were again the dependent variable and MFQ scores for the five moral domains were the predictor variables. The overall model did not significantly predict psychopathy, *R* ^*2*^ = .11 *F*(5,34) = 0.81, *p* = .550, and psychopathy was not predicted by judgments about causing harm, *β* = -.10, *t*(34) = 0.54, *p* = .595. The bivariate correlation between psychopathy and judgments about causing harm was also not significant, *r*(38) = -.13, *p* = .415, nor was the result of a median split for which scores on the harm domain of the MFQ were compared across participants with high and low psychopathy scores *t*(38) = 0.68, *p* = .499, *r* = .11. Responses to the remaining four moral domains also did not predict psychopathy. To assess validity of the MFQ in our sample, we calculated correlations between political orientation and the MFQ subscales, and found patterns that are consistent with previous reports [47]. Political liberalism was linked to more emphasis on Fairness, *r*(38) = -.35, *p* = .030, and Harm, *r*(38) = -.32, *p* = .050, and less emphasis on Authority, *r*(38) = .43, *p* = .006, and Purity *r*(38) = .30, *p* = .066. Psychopathy was not significantly related to political orientation, *r*(38) = -.23, *p* = .152. When we controlled for political orientation, psychopathic traits remained unrelated to judgments of harm on the MFQ, *β* = -.26, *t*(34) = 1.60, *p* = .119.

Finally, we conducted a multiple regression analysis to directly compare how judgments about causing fear (using the EEST) and judgments about causing harm (using the MFQ) predict psychopathy. The overall model was marginally significant, *R* ^*2*^ = .126, *F*(2,37) = 2.68, *p* = .082 (Table 2). Judgments about causing fear predicted psychopathy scores, *β* = .34, *t*(38) = 2.15, *p* = .038, whereas judgments about causing harm did not, *β* = -.05, *t*(38) = 0.34, *p* = .734.

**Table 2:** Regression model with moral evaluations of causing fear from the Emotionally Evocative Statements Task (EEST) and harm sensitivity from the Moral Foundations Questionnaire (MFQ) predicting psychopathy scores.

Predictor Variables	*β*	*t*(39)
**EEST Fear**	.34	2.15*
**MFQ Harm**	-.05	-0.34

Overall model: *R* ^2^ = .126, *F*(2, 37) = 2.68, *p* = .082.

* *p* < .05

To evaluate consistency with previous findings, we also analyzed patterns of emotion identification on the EEST. Results were largely consistent with previous findings [31]. Regression results showed that judgments about how fear-causing statements would make others feel were the strongest predictors of psychopathy, although only marginally significant, *r* = -.29, *p* = .074. Judgments about how fear-causing statements would make oneself feel were significant predictors of psychopathy, *r* = -.38, *p* = .017, as were moral judgments about causing happiness, *r* = -.39, *p* = .014, and sadness, *r* = .35, *p* = .025. As previously observed, we found that evaluations about how fear-causing statements would make oneself feel mediated the relationship between psychopathy and judgments about the moral acceptability of making these statements, Sobel *Z* = 2.02, *p* = .044.

### Discussion

This study compares the results of two validated moral judgment tasks: one that assesses moral judgments about causing others various forms of emotional distress, particularly fear, and another that focuses on moral judgments about causing others physical harm. Results were in line with predictions that psychopathy is more closely associated with judgments about the moral permissibility of causing others emotional distress, specifically fear, than judgments about causing harm. This is consistent with the hypothesis that individuals with high levels of psychopathic traits disproportionately engage in antisocial behaviors that cause others distress not simply because they are unaware of the physical harm that behaviors such as violent aggression may cause, but because they do not fully appreciate the emotional consequences of these actions for the victims [34,9]. Supporting the validity of our findings are several outcomes that replicate previous findings. Judgments about causing fear were more closely associated with psychopathy than judgments about causing any other emotion [31,32]. We also replicated previous findings regarding the relationship between moral foundations and political orientation [47].

It should be noted that previous research using a larger sample has found that the harm foundation of the MFQ is related to psychopathy in a regression model that controlled for multiple variables [53]. The present findings do not necessarily refute these prior findings, but rather demonstrate that within a given sample the relationship between psychopathy and judgments about causing harm is not as strong as the relationship between psychopathy and judgments about causing fear. However, the differences between the MFQ and the EEST instruments limit our ability to draw strong conclusions about the relative importance about judgments of fear versus harm in psychopathy from this study. The MFQ focuses on moral attitudes and the EEST focuses on socially embedded interpersonal interactions, such that these two measures may not be ideal for comparing sensitivity to the consequences of causing harm versus fear. Therefore, for Study 2 it was necessary that we develop a novel moral judgment task composed of scenarios modeled on the dilemmas previously used to assess moral judgments in psychopathy [35,40,54]. These scenarios were designed specifically to disambiguate judgments about causing others physical harm and emotional distress while controlling for other influences on moral judgments (e.g., number of victims, intentionality). In each scenario, participants judged the permissibility of actions that caused one of four outcomes: physical harm, emotional distress (fear), both harm and distress, or neither harm nor distress. Through the creation of the novel moral judgment task we aimed to avoid the limitations inherent in comparing the EEST and MFQ.

## Study 2

### Participants

Thirty-nine males (*N* = 10) and females, ages 18–22 years (*M* = 20.54, *SD* = 1.42) were recruited from the community and Georgetown University and participated in exchange for either monetary compensation or class credit. Written consent was obtained from all participants prior to participation in the study. No participants were excluded from analysis.

### Method

We generated four versions of each of eight novel moral dilemmas, for a total of 32 dilemmas. The four versions of each dilemma featured the same root scenario (e.g., being mocked by a passerby in a park) but systematically varied whether the victim in the scenario was physically harmed and whether the victim experienced emotional distress in anticipation of physical harm (i.e., experienced fear) (Table 3), in keeping with the conceptualization of fear as the emotional state that accompanies the anticipation of a possible aversive outcome [9]. Furthermore, the root action engaged in by the actor in each scenario was held consistent across all four versions of the scenarios (e.g., throwing a rock). Thus, the four versions of each dilemma featured, respectively, a victim who was physically harmed (but not frightened), who was frightened (but not harmed), who was harmed and frightened, or who was neither harmed nor frightened (neutral scenario). In each dilemma, the outcome of the action was always the intended outcome, such that the intention, action, and outcome (to harm or not harm, and/or to frighten or not frighten) were all mutually consistent in each dilemma.

**Table 3:** Sample scenarios.

**Root scenario**	**You are sitting in a public park when a person you don’t know walks by you. He stares at you as he walks by and then points at you and starts laughing. You are pretty sure he is making fun of you. There are several large rocks on the ground by your feet.**
***Variations***	
**Harm and Fear**	… As the stranger walks by you shout to get his attention and he turns to look at you. He flinches and cries out as you throw a rock at him as hard as you can. You hit him in the temple and he falls down, unconscious.
**Harm**	… You wait until after the stranger has walked past you a few feet. He is unaware as you throw a rock at him as hard as you can. You hit him in the temple and he falls down, unconscious.
**Fear**	… As the stranger walks by you shout to get his attention and he turns to look at you. He flinches and cries out as you throw a rock at him as hard as you can. You deliberately aim a little wide, the rock misses him by barely an inch, and he runs away.
**No Harm or Fear (Neutral)**	… You wait until after the stranger has walked past you a few feet. He is unaware as you throw a rock at him as hard as you can. You deliberately aim a little wide, the rock misses him by barely an inch, and he continues walking away without noticing.

Eight versions of the task were created, and in each participants read and responded to eight dilemmas total, including two dilemmas of each type, each of which featured a different root scenario. In this way, participants did not respond to any two dilemmas featuring the same root scenario. The order of the dilemmas within each task was randomized. During the task, participants read the dilemmas three times. In the first round, participants were instructed to, “decide how morally acceptable the behavior described in the scenario would be”. The second and third rounds were manipulation checks in which participants judged how frightened the victim would be and the extent to which the victim was harmed. All responses were collected using a 7-point response scale. The order of the second and third rounds was counterbalanced across participants, such that some participants judged harm before fear, and for others these judgments were reversed. Both types of judgments always followed judgments about moral permissibility so that permissibility judgments would not be affected by participants’ awareness that the study was assessing responses to causing physical harm versus emotional distress. Finally, participants completed the PPI-R and a demographics questionnaire.

We hypothesized that widespread semantic knowledge of proscriptions against causing physical harm would leave moral judgments in response to dilemmas featuring physical harm relatively unaffected by psychopathy, given evidence that high psychopathy scorers rely more heavily on semantic knowledge during moral decision making. By contrast, we hypothesized that when making judgments about causing emotional distress (fear) in the absence of physical harm, high psychopathy scorers would judge it more acceptable to cause others fear than would low scorers.

### Results

We first calculated PPI-R total scores for all participants. Scores ranged from 209–341 (*M* = 274.56, *SD* = 32.37). Scale reliability was acceptable (α = .85). Recalling our hypothesis that high psychopathy scorers will show impaired moral judgments when judgments about causing fear are dissociated from judgments about causing harm, we conducted a repeated measures GLM analysis examining judgments of moral acceptability to test the 3-way interaction among psychopathy, moral judgments about causing harm, and moral judgments about causing fear. The presence or absence of harm and fear were entered as dichotomous factors and psychopathy scores were entered as a continuous covariate. Results revealed the hypothesized three-way interaction between psychopathy, moral judgments about causing harm and moral judgments about causing fear, *F*(1,38) = 4.64 *p* = .038, *η*_*p*_^*2*^ = .111. In addition, a main effect of psychopathic traits was identified, *F*(1,38) = 10.24, *p* = .003, *η*_*p*_^*2*^ = .217, but no two-way interactions between psychopathy and judgments about causing harm or fear were identified. No significant main effects related to judgments of causing harm, *F*(1,38) = 0.44, *p* = .518, *η*_*p*_^*2*^ = .011, or fear, *F*(1,38) = 0.18, *p* = .675, *η*_*p*_^*2*^ = .005, emerged.

We considered two approaches to investigate the three-way interaction. First, we corrected for the main effect of psychopathy, according to which high psychopathy scorers tend to judge all scenarios, including neutral scenarios, to be more permissible [55], by calculating difference scores for responses to each dilemma versus neutral dilemmas. We then conducted linear regressions to examine linear relationships between psychopathy and moral judgments across conditions. Results showed no significant linear relationship between psychopathy and judgments of causing harm and fear, harm only, or fear only (all *p*s > .05) after correcting for the main effect of psychopathy.

These patterns may be consistent with the presence of non-linear effects across high and low psychopathy scorers due to qualitative differences in the moral reasoning strategies employed [32,43–45]. Therefore, to examine low and high scorers separately we performed a median split to create low psychopathy (*M* score = 249.75, *SD* = 23.67) and high psychopathy (*M* score = 298.12, *SD* = 19.13) groups [31, 56]. The 3-way interaction among psychopathy, moral judgments of harm and moral judgments of fear persisted following the median split, *F*(1,37) = 6.66, *p* = .014, *η*_*p*_^*2*^ = .153. We next collapsed scores across categories to compare average scores for dilemmas featuring harm versus no harm and to compare dilemmas featuring fear versus no fear and found that low psychopathy scorers judged causing harm to be worse than not causing harm, *t*(18) = 10.33, *p* < .001, *r* = -.78, and causing fear to be worse than not causing fear, *t*(19) = 5.64, *p* < .001, *r* = -.45. High psychopathy scorers also judged causing harm to be worse than not causing harm, *t*(19) = 6.73, *p* < .001, *r* = -.70, but they did not judge causing fear to be worse than not causing fear, *t*(19) = 1.29, *p* = .212, *r* = -.18. These results suggest that, consistent with the results of Study 1, psychopathy is not strongly predictive of moral judgments about causing harm. However, psychopathy scores are strong predictors of moral judgments about causing fear. This suggests that the strongest group differences will be observed in scenarios in which victims experience fear (but not harm). In addition, these effects emerge more strongly when high and low scorers are examined separately.

**Fig 1:**
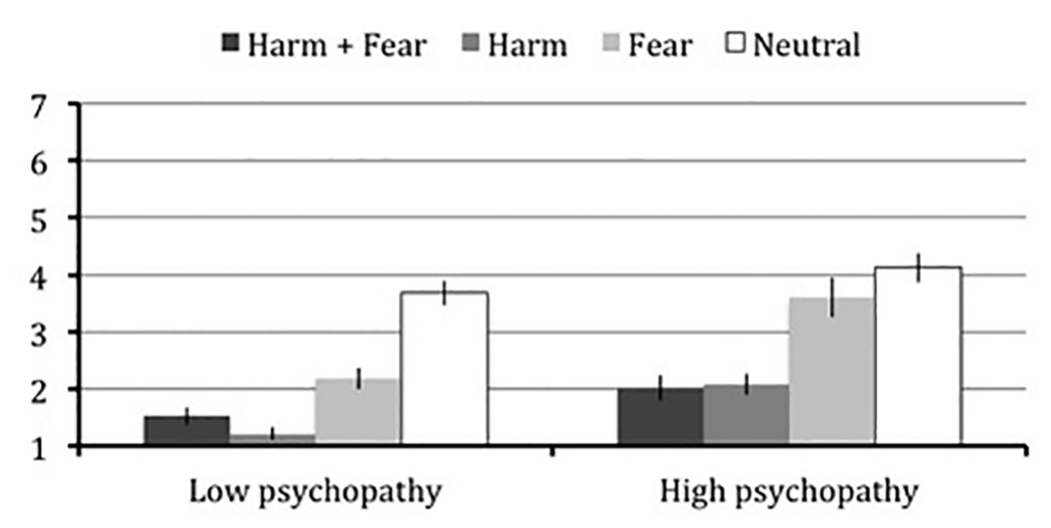
Study 2—Moral permissibility judgments in high and low psychopathy scorers. Mean and standard error from Study 2 for moral permissibility ratings of each of the four moral reasoning conditions with high and low psychopathy scorers, as determined by a median split, plotted separately.

We also examined responses to each of the four scenario types separately across groups (Fig 1). Low psychopathy scorers judged all dilemmas to be less morally permissible than the neutral (neither harm nor fear) dilemmas: harm and fear, *t*(19) = 11.28, *p* < .001, *r* = .80, harm only, *t*(19) = 11.18, *p* < .001, *r* = .86, and fear only, *t*(19) = 7.41, *p* < .001, *r* = .65. By contrast, high psychopathy scorers judged harm-based scenarios to be less morally permissible than neutral dilemmas (neither harm nor fear): harm and fear, *t*(19) = 6.57, *p* < .001, *r* = .69, harm only, *t*(19) = 6.40, *p* < .001, *r* = .71, but did not differentiate between fear-only and neutral dilemmas, *t*(18) = 1.44, *p* = .165, *r* = .19. Due to the observed main effect of psychopathy, *F*(1,37) = 16.18, *p* < .001, *η*_*p*_^*2*^ = .304, we calculated difference scores between responses to each dilemma and responses to neutral (no harm or fear) dilemmas to correct for this effect. Comparing the resulting scores across groups, we observed no group differences for moral judgments about causing harm and fear, *t*(37) = 0.15, *p* = .879, *r* = .02, or harm only, *t*(37) = 1.08, *p* = .288, *r* = .17. By contrast, high psychopathy scorers judged causing fear only to be a more morally acceptable course of action than did low scorers *t*(37) = 2.31, *p* = .027, *r* = .35. Examination of the subscale scores of the PPI-R revealed no statistically significant relationships between any of the subscales and moral judgments about causing fear in others. However, the Impulsive Antisociality subscale was most closely linked to moral judgments at a trend level, *r*(39) = .29, *p* = .069.

Analyses of participants’ judgments of the harm and fear experienced by the victim in each scenario confirmed that participants judged more harm to be occurring in harm scenarios, *F*(1,37) = 232.69, *p* < .001, *η*_*p*_^*2*^ = .863, and more fear occurring in the fear scenarios *F*(1,37) = 147.83, *p* < .001, *η*_*p*_^*2*^ = .800. No main effects or interactions related to psychopathy scores emerged (all *p*s > .10). Only one significant relationship emerged between subscales of the PPI-R and judgments of fear experienced by the victim. Fearless Dominance subscale scores were associated with judgments that victims experienced less fear in scenarios featuring fear, *r*(39) = -.36, *p* = .026. Table 4 contains means and standard deviations for perceptions of harm and fear for each condition.

**Table 4:** Descriptive statistics for evaluations of perceived harm and fear for each moral dilemma condition.

	Harm + Fear	Harm	Fear	Neutral
**Study 2**				
Harm Ratings	5.65 (1.12)	5.78 (1.07)	2.58 (1.45)	1.76 (0.99)
Fear Rating	5.64 (1.02)	3.56 (1.72)	5.31 (0.97)	2.17 (1.11)
**Study 3**				
Harm Ratings	5.78 (0.98)	5.77 (0.99)	2.29 (1.24)	1.99 (1.06)
Fear Rating	5.52 (1.07)	4.17 (1.95)	5.35 (1.14)	2.90 (1.47)

### Discussion

These results support our hypothesis that psychopathy affects moral judgments about causing emotional distress more strongly than judgments about causing physical harm. The three-way interaction revealed by our analyses is consistent with our hypothesis that moral judgments are affected most strongly by psychopathy when emotional distress is present but physical harm is absent. Judgments about the acceptability of physically harming another person in order to achieve a goal were similar across participants. But low psychopathy scorers judged causing someone to *fear* imminent harm (by, for example, threatening the person) to be less morally acceptable than high scorers. By contrast, high psychopathy scorers judged threatening injury to be morally indistinguishable from neutral scenarios (in which the victim was neither frightened nor harmed). This finding is consistent with a large number of previous findings that psychopathy impairs the ability to recognize or empathize with others’ experiences of fear [9] and suggests that it is high psychopathy scorers’ failure to understand fear as a deleterious experience in its own right that leaves them blind to the moral consequences of behaviors that cause others emotional distress. By contrast, high psychopathy scorers are equally likely as low scorers to judge causing others harm to be morally impermissible. Our findings that moral judgments about causing harm and fear, harm only, and fear only were not significantly linearly related to psychopathy are consistent with previous findings that, although psychopathic traits may be continuously distributed, the *expression* of these traits may not be. In particular, the relationship between psychopathy and moral reasoning may be discontinuous. In using a median split approach, our analyses allow us to identify differences in moral decision-making processes in high versus low psychopathy scores.

Our findings did not reveal significant group differences in the attribution of fear or harm caused to the victims in the various conditions. All participants perceived significantly greater levels of fear in scenarios intended to depict fear and significantly greater levels of harm in scenarios intended to depict harm. No effects of psychopathy were hypothesized in these analyses because the scenarios were not developed to test fear recognition, and judgments about causing harm and fear were intended primarily as manipulation checks. It was our explicit goal in designing the vignettes that the depicted fear be equally salient and interpretable as the depicted harm. Because the salience of both harm and fear was maximized by design, the dilemmas were likely not sufficiently sensitive to detect group differences in harm or fear recognition.

A question remaining is whether high psychopathy scorers’ impairments in judging the moral consequences of causing others emotional distress is related to distress-causing behaviors like aggression. Psychopathy is closely linked to proactive antisocial behaviors that victimize others to achieve instrumental gain [5,57]. In Study 3, we aimed to replicate our findings from Study 2 and link them to a measure that indexes both proactive aggression and reactive aggression in daily life and has been demonstrated to discriminate these forms of behavior in psychopathy [58]. We predicted that proactive aggression would be associated with both psychopathy and impaired moral judgments about causing others distress, but that reactive aggression would not show these associations.

## Study 3

### Participants

Fifty-two participants were recruited from Georgetown University and the surrounding community and participated in exchange for either monetary compensation or class credit. Written consent was obtained from all participants prior to participation in the study. Data from one participant were removed because the participant entered identical responses across all questions. The remaining fifty-one participants, males (*N* = 13) and females, were ages 18–37 years (*M* = 21.67, *SD* = 3.24).

### Method and Results

Participants completed the experimental task described in Study 2 and the PPI-R, as well as the Reactive-Proactive Aggression Questionnaire (RPQ) [58]. The RPQ is a 23-item self-report questionnaire that assesses physically and verbally aggressive proactive and reactive aggression behaviors, including bullying, yelling to intimidate others, stealing, and tantrums. Items are coded on a 3-point scale (0 = Never, 1 = Sometimes, 2 = Often). The RPQ is a self-report measure that exhibits excellent construct and predictive validity, with the proactive subscale predicting objectively measured conduct problems and externalizing behaviors [59] and initiation of fights, delinquency, serious violent offending and psychopathic traits in adolescence [58].

PPI-R scores ranged from 184–353 (*M* = 262.47, *SD* = 41.26). Scale reliability was acceptable (α = .78). RPQ scores ranged from 0–31 (*M* = 9.78, *SD* = 5.53) and scale reliability was again acceptable (α = .84). Following our analytic strategy in Study 2, we replicated results from Study 2, finding a significant 3-way interaction among psychopathy, harm and fear through the GLM analysis examining judgments of moral acceptability with psychopathy entered as a continuous covariate and the presence of harm or fear entered as dichotomous factors, *F*(1,49) = 5.38, *p* = .025, *η*_*p*_^*2*^ = .099. Consistent with Study 2, after correcting for the main effect of psychopathy, *F*(1,49) = 5.31, *p* = .025, *η*_*p*_^*2*^ = .098, regression analyses revealed a non-significant linear relationship between psychopathy and causing harm and fear, harm only or fear only (all *p*s > .05).

**Fig 2:**
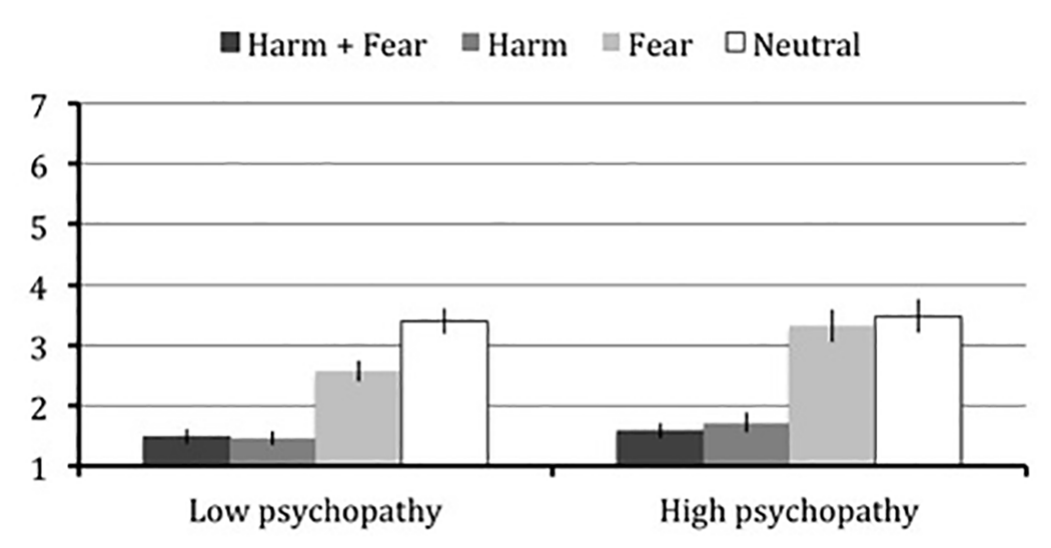
Study 3—Moral permissibility judgments in high and low psychopathy scorers. Mean and standard error from Study 3 for moral permissibility ratings of each of the four moral reasoning conditions with high and low psychopathy scorers, as determined by a median split, plotted separately.

Next, we again used a median split (median score = 258) to create a low psychopathy (*M* score = 229.65, *SD* = 21.00) and a high psychopathy (*M* score = 296.62, *SD* = 26.61) group. We again found the hypothesized significant 3-way interaction among psychopathy, harm, and fear, *F*(1,49) = 4.63, *p* = .036, *η*_*p*_^*2*^ = .086, with low psychopathy participants once more judging all scenario types to be less morally acceptable than neutral: harm and fear, *t*(25) = 7.36, *p* < .001, *r* = .73; harm only, *t*(25) = 9.09, *p* < .001, *r* = .75; fear only, *t*(25) = 3.88, *p* < .001, *r* = .39. By contrast, high psychopathy participants judged only harm-based scenarios to be less morally acceptable than neutral: harm and fear, *t*(24) = 5.94, *p* < .001, *r* = .66; harm only, *t*(24) = 6.50, *p* < .001, *r* = .61. And again, high psychopathy scorers did not distinguish causing fear only from neutral scenarios, *t*(24) = 0.67, *p* = .543, *r* = .06 (Fig 2).

A marginally significant main effect of psychopathy was again found, *F*(1,49) = 2.91, *p* = .094, *η*_*p*_^*2*^ = .056, whereby high psychopathy scorers judged all scenarios (including neutral) to be more permissible, and for consistency we corrected for this main effect. No group differences emerged for moral judgments about causing harm: harm and fear, *t*(49) = 0.06, *p* = .956, *r* = .01, harm only, *t*(49) = 0.53, *p* = .598, *r* = .07. But high psychopathy participants judged causing others fear to be more morally permissible than low psychopathy participants at a trend level, *t*(49) = 1.99, *p* = .052, *r* = .27.

**Fig 3:**
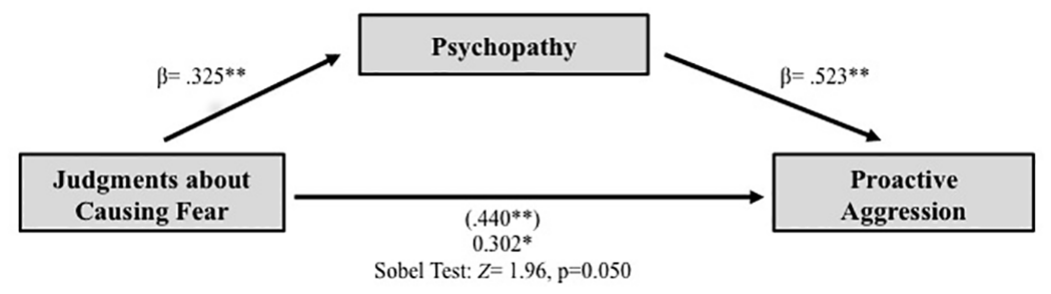
Psychopathy mediates the relationship between distress sensitivity and proactive aggression. Increased psychopathic traits mediate the relationship between impaired moral judgments of causing fear and increases in proactive aggression.

Finally, we observed the hypothesized group differences in self-reported aggression as measured using the RPQ, with high psychopathy scorers reporting engaging in more aggression, *t*(49) = 2.41, *p* = .020, *r* = -.32. This effect was accounted for by differences in proactive aggression, *t*(49) = 3.50, *p* = .001, *r* = -.44. No group differences in reactive aggression emerged, *t*(49) = 1.12, *p* = .268, *r* = -.15. In addition, judgments about the permissibility of causing fear in the moral judgment task predicted proactive aggression, *r*(51) = .44, *p* = .001 but not reactive aggression, *r*(51) = .20, *p* = .168. A multiple regression analysis including both reactive and proactive aggression as predictor variables of the moral permissibility of causing fear confirmed that proactive aggression, *β* = .43, *t*(49) = 3.04, *p* = .004, but not reactive aggression, *β* = .02, *t*(49) = .12, *p* = .907, is specifically linked to abnormal moral judgments about causing fear. We therefore conducted a Sobel test of mediation examining the extent to which psychopathy influences judgments about causing fear and proactive aggression. Unstandardized regression coefficients and standard errors were entered into the model for each path to determine if the indirect effect of sensitivity to fear on proactive aggression through psychopathy is significantly different from zero [60]. Results showed that psychopathy mediated the relationship between moral judgments about causing fear and proactive aggression, Sobel *Z* = 1.96, *p* = .050 (Fig 3). By contrast, moral judgments about causing fear did not mediate the relationship between psychopathy and reactive aggression. The mediation pathway is consistent with the hypothesis that latent individual variation in fear responsiveness results in psychopathic personality traits that increase risk for proactive aggression [31].

The relationships among subscale scores of the PPI-R, moral judgments, and aggression were examined. Results revealed significant relationships between the Impulsive Antisociality subscale and both moral judgments about causing others fear and proactive (but not reactive) aggression. Individuals with high scores on the Impulsive Antisociality subscale judged causing others fear to be more morally acceptable, *r*(51) = .38, *p* = .006, and also reported higher levels of proactive aggression, *r*(51) = .53, *p* < .001. Consistent with results for the total PPI-R, a Sobel mediation analysis showed that Impulsive Antisociality mediated the relationship between moral judgments about causing fear and proactive aggression, Sobel *Z* = 2.42, *p* = .019.

We also found again that participants judged more harm to be occurring in harm scenarios, *F*(1,49) = 1390.41, *p* < .001, *η*_*p*_^*2*^ = .902, and more fear to be occurring in fear scenarios, *F*(1,49) = 70.14, *p* < .001, *η*_*p*_^*2*^ = .589. Again, there were no significant main effects or interactions related to psychopathy scores (all *p*s > .10). No significant results emerged through the examination of subscales of the PPI-R. Means and standard deviations for perceptions of harm and fear for each moral dilemma condition can be found in Table 4.

We also considered the role of gender in the findings from Studies 2 and 3. Due to low numbers of males in these studies, we combined data from both studies to generate sufficient power to enter gender in as an interacting factor. The combined sample consisted of 67 females and 23 males. We again conducted a GLM analysis examining judgments of moral permissibility with the presence or absence of harm and fear entered as dichotomous factors and psychopathy entered as a continuous variable. Results revealed no main effect of gender *F*(1,87) = 0.57, *p* = .451, *η*_*p*_^*2*^ = .007. Furthermore, our hypothesized 3-way interaction remained essentially unchanged after controlling for gender, *F*(1,87) = 4.92, *p* = .029, *η*_*p*_^*2*^ = .054. We again found a significant main effect of psychopathy when controlling for gender, *F*(1,87) = 15.47, *p* < .001, *η*_*p*_^*2*^ = .151, and therefore corrected for the main effect of psychopathy following the same procedures previously described. Next, we conducted a univariate GLM to test for the effects of gender on group differences in judgments about the moral acceptability of causing others fear only. High and low psychopathy scores were entered as a fixed factor and gender was entered as a covariate. We found that gender had a nonsignificant relationship with moral judgments of causing fear only, *p* > .050, while psychopathy continued to be a significant predictor, *F*(1,87) = 6.84, *p* = .010, *η*_*p*_^*2*^ = .073. Neither psychopathy nor gender was significantly related to moral judgments of causing harm and fear or harm only (all *p*s > .10).

### Discussion

Psychopathy is notoriously associated with pervasive immoral and antisocial behavior [57]. This relationship was confirmed in the present research, with high psychopathy participants in our community samples reporting engaging in behaviors like threatening, bullying, and using force to obtain money or goods. Understanding why individuals with psychopathic traits engage in these behaviors is a question of ongoing concern. One prominent theory is that basic affective deficits in psychopathy prevent individuals with psychopathic traits from learning that behaviors that cause others distress should be avoided [34]. The results of some moral reasoning paradigms in psychopathy lend support to this theory [31,32,35,37]. But other studies have failed to find that psychopathy impairs judgments about harming victims using standard moral dilemmas [40,42,41]. We argue that this is because standard moral dilemmas are designed to test abstract principles of moral reasoning rather than being designed to isolate judgments about causing victims distress, and that reliance on semantic reasoning is insufficient to generate a moral response in the absence of the appropriate affective processes. No previous study has explicitly separated judgments about causing physical harm from judgments about causing emotional distress. In the current study, we aimed to explicitly separate these types of judgments and found that psychopathy significantly affects judgments about causing others emotional distress while having relatively less impact on judgments of physical harm

We first demonstrated this using two previously validated tasks that separately assess moral judgments about causing harm and fear. In addition, we created a novel task for which the scenarios were closely modeled on the structure of scenarios used to assess moral judgments in psychopathy previously [40,42,41,54]. However, the 32 scenarios we generated were specifically designed to disambiguate judgments about causing harm from judgments about causing emotional distress (fear, specifically). This is in contrast to most moral dilemmas, which do not generally manipulate or even describe the emotional responses of victims to the harm that befalls them. Our results showed that whereas psychopathic traits are not a reliable predictor of moral judgments about causing physical harm, they reliably predict judgments that causing others fear is morally permissible. Importantly, moral judgments about causing others fear predict not only psychopathy, but also self-reported proactive aggression, which is the form of aggression most closely linked to psychopathy, whereby people engage in instrumental behaviors that cause others distress. Our finding that psychopathy mediates the relationship between moral judgments about causing fear and proactive aggression is consistent with the idea that etiologically, psychopathy emerges from impairments in the neurocognitive systems that support fear responding, which in turn results in a higher risk of proactive aggressive behavior [61].

Our finding that psychopathy especially impairs moral judgments in response to victims’ fear is significant. One of the most durable findings in the empirical literature on psychopathy is that it impairs the experience of fear [11–16]. In addition, psychopathy impairs the ability to recognize when another person is experiencing fear on the basis nonverbal cues expressed by the face, body, and voice [23]. Impaired fear recognition persists even for pre-attentively processed stimuli [1] and verbally presented stimuli [31], and is related to the callous-unemotional factor of psychopathy more strongly than the antisocial behavior factor [24]. Together, these findings suggest that a muted capacity for emotional distress in psychopathy may render individuals with psychopathic traits insensitive to the distress of victims and unable to generate an appropriate empathic reaction [62]. With reference to the present findings, we suggest that psychopathy is most likely to impair moral judgments when those judgments require the generation of an empathic response to a victim’s distress.

Many moral judgments do not require reference to victim distress. Consensus is emerging that there is not one neurocognitive system for generating moral judgments, but a plurality [63,64]. Judgments about the appropriateness of harming innocent victims can be achieved by considering the effects of harmful actions on the victim, but can also be achieved with reference to, for example, semantic information about societal rules about harming others [41]. The theory that high psychopathy individuals preferentially employ deliberate rule-based judgments during moral decision tasks is supported by the results of neuroimaging studies that find increased activation in dorsolateral prefrontal cortex in psychopathic respondents during morally relevant judgments, for example, trolley car dilemmas [41], judgments about causing others negative emotions [32] and the prisoner’s dilemma [46]. The dorsolateral prefrontal cortex is involved in facilitating abstract reasoning, specifically the capacity to represent and integrate complex relationships among stimuli during problem-solving tasks [65]. Activation in this region suggests that high psychopathy respondents preferentially recruit semantic information about societal rules to answer questions about moral permissibility, which may help to explain why high psychopathy participants have been observed to judge moral and conventional violations in similar ways [35,37]. It also may help to explain why psychopathic individuals persist in immoral behaviors that they recognize to be morally wrong. Presumably, simple semantic recognition of a behavior’s wrongfulness is an insufficient motivator to avoid the behavior if it may result in instrumental gain [41,44].

When no differences are observed between high and low psychopathy participants in a moral judgment tasks, it suggests that semantic or similarly deliberate cognitive strategies may be the default strategy across groups, or that similar judgments can be generated using either semantic or affectively-based strategies. Given the similar results observed across groups in our study during judgments of scenarios featuring physical harm, it is possible that these scenarios predominantly elicited semantically or cognitively based judgments across participants or that reliance on semantic or cognitive strategies of evaluating harm are sufficient to produce the same moral response than the additional employment of affectively-based strategies. This is consistent with the fact that proscriptions against causing others physical harm are highly familiar, more so than proscriptions against causing others to fear being harmed—although these behaviors are distinct and are *both* legal violations. Causing physical harm meets the legal definition of battery, and causing another to fear being harmed meets the definition of assault [66]. As a result, individuals with high levels of psychopathic traits may effectively employ semantic strategies when evaluating moral acceptability of causing physical harm due to the clear societal labeling of causing harm as morally wrong. One way to test this hypothesis could be to assess difference in response times to different scenarios across groups, as cognitively based strategies generally require longer response latencies than affective strategies [64,67]. One limitation of the present data is that Studies 2 and 3 were completed using paper and pencil rather than computers, preventing assessment of response times. Future computer-based research might enable this question to be addressed. Another strategy might be to assess how moral judgments are disrupted in high psychopathy and low psychopathy participants in the presence of affective or cognitive loads.

Future testing might also help to address an issue that arose following the design of the current Studies 2 and 3, in which the scenarios were aimed to make the harm and/or fear befalling the victims maximally salient. As a result, and as intended, psychopathy was unrelated to judgments about when fear was occurring even while psychopathy was associated with deficits in moral judgments about causing fear. This suggests that being able to identify the occurrence of fear is not in itself sufficient for generating normative moral judgments about causing fear among high psychopathy participants. Why might this be? Again, the answer may reflect the fact that respondents can use either semantic or affective strategies to respond to questions (regarding either the emotional content or the moral acceptability) about moral scenarios. When considering whether each scenario evoked fear, participants could rely on affective processes or semantic knowledge about when fear occurs. Because the question was simple—given the deliberate salience of the fear and the fact that only one emotion response category was available—either strategy would likely yield the correct answer. If, however, alternative response options had been available, the increased task difficulty may have made semantic knowledge insufficient for answering the question and yielded a stronger relationship between psychopathy and judgments of the occurrence of fear. This is akin to results in tasks where fear must be recognized using relatively ambiguous nonverbal cues such as facial expressions or posture [20,23] or brief verbal statements [31,32], which cannot easily be answered using semantic information. If our observed patterns of results were caused by the fear recognition task being overly simple, enabling even high psychopathy scorers to identify when fear would likely occur from the details supplied in our moral scenarios, we could conclude that simple semantic knowledge that fear is occurring is insufficient for generating normative moral judgments, which require lower-level affective processes.

It should be noted that a possible alternate reason that judgments about fear were unrelated to psychopathy in our paradigm is because in asking about victims’ fear, our paradigm induced participants to focus their attention on the fear-inducing aspects of the scenarios. Some theories suggest that psychopathy is primarily a deficit of attention, and that affective responses to fear-related stimuli can be normalized if attention is focused primarily on those stimuli [68,69,70]. If this were the case, it would suggest that high psychopathy scorers *can* recognize when fear is occurring in some cases, but may not do so automatically. In this case, we would expect that asking participants’ about the victims’ fear before asking them to make moral judgment might normalize high psychopathy participants’ moral judgments.

Adjudicating among these possible relationships among fear recognition, moral judgments, and psychopathy may not be possible using the present task design. To further explore the relationship between recognizing the occurrence of fear and making moral judgments about causing fear, pictorial stimuli featuring a victim about to be harmed or not, and expressing fear or not, might be useful. This format would allow the use of eye-tracking stimuli to record attention to the various features of the scenario (impending harm, the victim’s fear) in order to explore the role of attention in participants’ evaluations of emotion and moral judgments. The addition of a cued attention feature could assess the role of attention even more directly. Pictorial stimuli would also require participants’ to detect fear using more ambiguous nonverbal cues, which, coupled with multiple response options about the emotion depicted, might reduce the accuracy of semantically based judgments and generate increased variance in participants’ responses. Psychophysiological responses (such as skin conductance) might also be measured to more directly assess emotional responses to various categories of stimuli and allow for more direct assessment of the role of emotion in moral judgments about harm and emotional distress.

Other features of moral dilemmas that can affect moral judgments, such as intentionality or outcome, were not assessed in the present studies. The novel moral reasoning task used for Studies 2 and 3 did not explicitly instruct participants to attend to the intention or outcome present in each scenario. As a result, we cannot definitively determine whether or not individuals with high psychopathy scores are less effectively employing the same moral reasoning processes when evaluating causing others fear, or whether they are employing different moral reasoning processes than individuals with low psychopathy scores altogether. Investigation of the effects of directed attention towards intentions and outcomes of these moral dilemmas as well as investigation of the neural correlates could help to further illuminate the mechanisms that underlie moral judgments about causing others fear. Future research might also consider the possible role of ceiling effects in judgments of harm in the present studies. While the harm depicted in the current scenarios is less extreme than the harm depicted in commonly used moral dilemmas, such as trolley dilemmas in which the victims typically die, the clear and salient harm we aimed to depict could have resulted in reduced variance in participants’ moral judgments and/or ceiling effects.

An important consideration in interpreting the present data is that they were acquired using a community sample providing self-report measures of psychopathy rather than an institutionalized sample using file-data based assessments of psychopathy. Psychopathy is thought to be a continuously distributed trait in the general population rather than being taxonomic in structure [2,3], as is the case for other affective variables, which, in their most severe forms, are linked to psychopathology [71,72]. It should also be noted that our samples were composed of both males and females, and each sample contained more females than males. These samples are quite similar to the undergraduate samples also containing more females than males in which original PPI was developed and standardized [73], supporting the validity of these samples for understanding the nature of psychopathy using the PPI-R. Nonetheless, we conducted analyses testing for the possible confounding effects of gender on our results and found no main effect of gender on judgments of moral permissibility or judgments of causing harm and fear, harm only or fear only. Additionally, after controlling for gender, our hypothesized 3-way interaction remained significant and high and low psychopathy scores continued to predict judgments of causing others fear only but not harm and fear or harm only. Supporting the view that the factor structure and external correlates of most psychopathy measures has been found to be similar for males and females [74], our results do not seem to be significantly affected by the gender of our participants.

It would nevertheless be useful to confirm that the present patterns of results emerge in samples assessed using measures designed for institutionalized samples with higher base rates of antisocial behavior, such as a variant of the Psychopathy Checklist. We anticipate that findings in an institutionalized sample would be similar to those found in the community sample we used here, as previous moral judgment and related neurocognitive tasks have observed highly comparable findings across the psychopathy spectrum, whether using community samples or institutionalized samples [53,78–80]. Testing institutionalized samples using clinical measures would also enable further understanding of how the factors that compose the psychopathy construct relate to the observed moral judgments, as disagreements persist regarding how the factors of the PPI-R map onto those obtained using other measures of psychopathy [75–77]. Debate persists regarding the interpretation of the major subscales of the PPI-R [76,77] and thus, to maximize consistency with previous studies of moral judgments and emotion processing in psychopathy, which largely focus on the overarching construct of psychopathy, we focused on total PPI-R scores rather than on subscale scores.

## Conclusions

In describing the prototypical psychopath, Cleckley argued that, “He does not seem to intend much harm. In the disaster he brings about *he cannot estimate the affective reactions of others which are the substance of the disaster* [italics added]… the real psychopath seems to lack understanding of the nature and quality of the hurt and sorrow he brings to others” [81]. The present findings support this observation. We find that whereas psychopathy is minimally associated with moral judgments about causing others harm, it is more strongly associated with moral judgments about causing others distressed affective reactions, namely fear. This is consistent with the idea that deficits in sensitivity to others’ fear and distress are central to psychopathy and underlie the behavioral abnormalities that are characteristic of psychopathic individuals. In addition to illuminating the nature of moral dysfunction in psychopathy, these results also suggest that, more generally, moral judgments about causing victims distress and harm are dissociable, and that how we judge others’ *psychological* states—like fear—may be as critical or more critical than how we judge *physical* states—like harm—in understanding some aspects of moral judgments, empathy, and aggression.

## Supporting Information

S1 Text Complete set of moral dilemmas used in Studies 2 & 3. (DOC)
