# Supplementary material for: Impact of Psychopathy on Moral Judgments about Causing Fear and Physical Harm
Source: PLoS One. 2015 May 20;10(5):e0125708. doi: 10.1371/journal.pone.0125708 (PMC4438873; doi:10.1371/journal.pone.0125708)
Supplement: S1 Text — (DOC) [file pone.0125708.s001.doc]

**S1 Text*.* Complete set of moral dilemmas used in Studies 2 & 3**

| ***Root Scenario*** | You are a studying for a final exam and need an A to pass the class. Your roommate knows you are studying but has been talking loudly on the phone for hours, ruining your concentration. Finally, you are fed up and grab a heavy textbook off your desk and walk over to her, raising the textbook over your head... |
| --- | --- |
| ***Variations:***  *Harm and Fear* | … She sees you and flinches but doesn’t move her hand in time as you slam the textbook down on her hand that is holding the phone, causing her drop her phone |
| *Harm* | … She doesn’t see you and so doesn’t move her hand as you slam the textbook down on her hand that is holding the phone, causing her drop her phone. |
| *Fear* | … She flinches as you slam the textbook down an inch from her hand, deliberately missing her, causing her to drop her phone. |
| *No Harm or Fear (neutral)* | … She doesn’t see you as you use the textbook to push the phone out of her hand, causing her to drop her phone. |
| ***Root Scenario*** | You are in a movie theater on opening night of a film you have wanted to see for months. Right next to you is a theatergoer whose phone rings, and he picks it up and begins talking and laughing with the caller. You can hardly hear the movie… |
| ***Variations:***  *Harm and Fear* | … So you tap him on the arm, and hold the coffee you just purchased over his lap. He tries to leap out of the way, but you pour the entire cup of scalding liquid in his lap and he drops his phone. |
| *Harm* | … So without his noticing, you pick up the cup of coffee you just purchased, lean over and pour the entire cup of scalding liquid in his lap and he drops his phone. |
| *Fear* | … You tap him on the arm, holding up the coffee you just purchased, making it look like you are going to pour the entire cup of scalding liquid in his lap. You don’t, but he tries to leap out of the way and he drops his phone. |
| *No Harm or Fear (neutral)* | … You bump his arm as you reach down to pick up the cup of coffee you just purchased, and as he moves out of the way he drops his phone. |
| ***Root Scenario*** | You are out biking on a trail so crowded that anyone who swerves suddenly risks being hit by other bikers. You are moving fast and round a corner and see a jogger coming toward you, blocking your path. You have no time to stop, so you continue ahead… |
| ***Variations:***  *Harm and Fear* | … She shrieks as you aim straight for her but she can’t get out of the way in time and you and collide with her, knocking her to the ground into the path of several other bicycles. |
| *Harm* | … She doesn’t see you as you approach and so doesn’t move out of the way and you collide with her, knocking her to the ground into the path of several other bicycles. |
| *Fear* | … She shrieks as you aim straight for her as though you are going to collide with her, and she leaps off the trail at the last minute and you narrowly avoid her. |
| *No Harm or Fear (neutral)* | … She doesn’t see you coming, but she edges aside at the last minute and you narrowly avoid her. |
| ***Root Scenario*** | You are playing tag football and the other team is one point away from winning. A player on the other team is about to catch the winning pass. He reaches out to catch the ball as it sails toward him… |
| ***Variations:***  *Harm and Fear* | … He looks away from the ball and sees you lunge at him. He gasps and tries to jump out of the way, but you elbow him in the stomach as hard as you can and he drops the ball. |
| *Harm* | … He is focusing on the ball so he is taken unawares as you lunge at him and elbow him in the stomach as hard as you can, and he drops the ball. |
| *Fear* | … He looks away from the ball and sees you lunge at him. He gasps and tries to jump out of the way, as you pretend you’re about to elbow him in the stomach as hard as you can and he drops the ball. |
| *No Harm or Fear (neutral)* | … He doesn’t look away from the ball so is unaware that you are lunging toward him. As you run past, you tap the ball just before he catches it, and he drops the ball. |
| ***Root Scenario*** | You are running late for work and arrive at the bus stop just as the bus is about to pull away. A woman is walking away from you down the sidewalk and is between you and the closing bus door… |
| ***Variations:***  *Harm and Fear* | … She shrieks as you run toward her and shove her hard enough to knock her out of the way. She falls to the ground as you jump past her onto the bus, and the doors close behind you. |
| *Harm* | … She doesn’t see you run toward her and shove her hard enough to knock her out of the way. She falls to the ground as you jump past her onto the bus, and the doors close behind you. |
| *Fear* | … She shrieks as you run toward her as though you were going to shove her. She scrambles out of the way as you jump past her onto the bus, and the doors close behind you. |
| *No Harm or Fear (neutral)* | … She doesn’t see you as you run toward her. She is walking just fast enough that there is room for you to jump past her onto the bus, and the doors close behind you. |
| ***Root Scenario*** | You are shopping the Black Friday sales at the mall after Thanksgiving. You have your heart set on a limited availability necklace for your mother. When you walk into the store that carries it, you see another customer reaching for it… |
| ***Variations:***  *Harm and Fear* | … You run towards her, shouting, and she turns and sees you and flinches just before you grab her arm, pulling her into a large display shelf, causing boxes to fall down onto her. You grab the necklace and head for the cash registers. |
| *Harm* | … You run up behind her silently and she doesn’t see you coming as you as you grab her arm, pulling her into a large display shelf, causing boxes to fall down onto her. You grab the necklace and head for the cash registers. |
| *Fear* | … You run towards her, shouting, and she turns and sees you and flinches, scrambling out of the way and knocking the necklace onto the ground. You grab the necklace and head for the cash register. |
| *No Harm or Fear (neutral)* | … You run towards her and she doesn’t see you as you edge past her, pushing the necklace onto the ground and out of her reach. You grab the necklace and head for the cash register. |
| ***Root Scenario*** | You are sitting in a public park when a person you don’t know walks by you. He stares at you as he walks by and then points at you and starts laughing. You are pretty sure he is making fun of you. There are several large rocks on the ground by your feet... |
| ***Variations:***  *Harm and Fear* | … As the stranger walks by you shout to get his attention and he turns to look at you. He flinches and cries out as you throw a rock at him as hard as you can. You hit him in the temple and he falls down, unconscious. |
| *Harm* | … You wait until after the stranger has walked past you a few feet. He is unaware as you throw a rock at him as hard as you can. You hit him in the temple and he falls down, unconscious. |
| *Fear* | … As the stranger walks by you shout to get his attention and he turns to look at you. He flinches and cries out as you throw a rock at him as hard as you can. You deliberately aim a little wide, the rock misses him by barely an inch, and he runs away. |
| *No Harm or Fear (neutral)* | … You wait until after the stranger has walked past you a few feet. He is unaware as you throw a rock at him as hard as you can. You deliberately aim a little wide, the rock misses him by barely an inch, and he continues walking away without noticing. |
| ***Root Scenario*** | You’re leaving a sports game that your team has just lost, and pass a group of fans celebrating the other team’s win. As your car approaches them, you roll down your window… |
| ***Variations:***  *Harm and Fear* | … and honk at them. As they turn to look at you, you throw a glass mug out the window at one of them, who sees you but can’t dodge out of the way in time. He crouches and tries to cover his head and the mug smashes into his forehead as you roll up your window and drive away. |
| *Harm* | … They don’t turn around, and you throw a glass mug out the window at one of them, who doesn’t see you and so doesn’t dodge out of the way. The mug smashes into his forehead as you roll up your window and drive away. |
| *Fear* | … and honk at them. As they turn to look at you, you throw a glass mug out the window toward one of them, deliberately aiming at a chair behind him. The mug barely misses him as he crouches and covers his head and you roll up your window and drive away. |
| *No Harm or Fear (neutral)* | … They don’t turn around, and you throw a glass mug out the window at one of the chairs near the group. The mug smashes into it without their noticing and you roll up your window and drive away. |
